# The ethics of forgoing life-sustaining treatment: theoretical considerations and clinical decision making

**DOI:** 10.1186/2049-6958-9-14

**Published:** 2014-03-11

**Authors:** Jos VM Welie, Henk AMJ ten Have

**Affiliations:** 1 Center for Health Policy and Ethics, Creighton University, 2500 California Plaza, Omaha NE 68178, USA; 2 Center for Healthcare Ethics, Duquesne University, Fisher Hall 300, 600 Forbes Avenue, Pittsburgh, PA 15282, USA

**Keywords:** Clinical ethics, Ethical theory, Euthanasia, Forgoing treatment, Life-sustaining treatment, Physician assistance in suicide, Ventilation, Withdrawing treatment, Withholding treatment

## Abstract

Withholding or withdrawing a life-sustaining treatment tends to be very challenging for health care providers, patients, and their family members alike. When a patient’s life seems to be nearing its end, it is generally felt that the morally best approach is to try a new intervention, continue all treatments, attempt an experimental course of action, in short, do something. In contrast to this common practice, the authors argue that in most instances, the morally safer route is actually to forgo life-sustaining treatments, particularly when their likelihood to effectuate a truly beneficial outcome has become small relative to the odds of harming the patient. The ethical analysis proceeds in three stages. First, the difference between neglectful omission and passive acquiescence is explained. Next, the two necessary conditions for any medical treatment, i.e., that it is medically indicated and that consent is obtained, are applied to life-sustaining interventions. Finally, the difference between withholding and withdrawing a life-sustaining treatment is discussed. In the second part of the paper the authors show how these theoretical-ethical considerations can guide clinical-ethical decision making. A case vignette is presented about a patient who cannot be weaned off the ventilator post-surgery. The ethical analysis of this case proceeds through three stages. First, it is shown that and why withdrawal of the ventilator in this case does not equate assistance in suicide or euthanasia. Next, the question is raised whether continued ventilation can be justified medically, or has become futile. Finally, the need for the health care team to obtain consent for the continuation of the ventilation is discussed.

## Theoretical considerations - Part l

### Introduction

One of the ethically most vexing decisions for clinical care providers is to withdraw a life-sustaining treatment. Many of the hallmark cases in American bioethics involve exactly that type of decision. In the case of Ms. Karen Quinlan [1], which is now half a century old, the treatment forgone was ventilation. Mr. Cinque refused continued dialysis [2]. Mr. Dax Cowart refused further treatment of his life-threatening burns [3]. The husband of Ms. Terry Schiavo wanted the artificial nutrition and hydration stopped after his wife had been in a persistent vegetative state (PVS) for more than two years [4]. All of these cases ended up in court. And when, more recently, a nurse at a California nursing home refused to provide cardio-pulmonary resuscitation (CPR) in accordance with the facility’s Do-Not-Resuscitate (DNR) policy [5], many a commentator was appalled.

Why the upheaval about the events in the nursing home? After all, the success rates of CPR, particularly for a frail 87 year old nursing home resident, are abominable [6,7] and the side-effects frequent and significant [8]. Even when it became clear from testimony of the family that the patient had been aware of the facility’s DNR policy and did not want CPR, some commentators continued to insist that the facility’s staff should have attempted CPR anyway, as was done by the Emergency Medical Services personnel upon their arrival at the scene – to no avail.

This insistence on CPR reflects a widespread and deep seated angst about withholding or withdrawing any type of life-sustaining intervention. Although most professional care givers are aware of the potential harm that can come to their patients when certain life-sustaining treatments are attempted or continued, many assume that the morally safer route is to always provide the treatment rather than withhold or withdraw it [9]. But is it?

In this article we will argue that the aforementioned assumption is mistaken. We will show that in most instances, the morally safer route is to forgo life-sustaining treatments, particularly when their likelihood to effectuate a truly beneficial outcome has become small relative to the odds of actually harming the patient. We will argue that the burden of proof and justification does not rest on the health professional who wants to withhold or withdraw a life-sustaining treatment, but rather on the one who wants to initiate or continue such treatment. From an ethical perspective, the default is “do *not* treat.”

### To act or not to act

Medicine’s preoccupation with *non*-treatment decisions seems unusual when compared with most other scenarios in life in which we face some kind of moral quandary. The designated driver does not run a moral risk if he decides *not* to accept the drink offered to him by a dear friend, but only when he *does* accept. If he caves in and accepts the drink, this moral risk materializes when he next gets into his car and drives himself and the members in his party back home. It is by *doing* certain things, by *bringing about* some kind of change in the natural course of events, that we become morally responsible.

This is why a thoracic surgeon about to embark on a lung transplant must carefully assess whether that modification of the patient’s body is beneficial to the patient before starting the intervention; this is why she must first obtain the patient’s consent to the surgery. In contrast, if the surgeon were to decide that a transplant is too dangerous for this frail patient, and hence rules out that treatment option, she does not need the patient’s consent *not* to transplant.

Now there are situations in which we incur moral responsibility even if we did not do anything. Precisely because we have the freedom to act or not to act, to intervene or stand by, to assist or ignore, we can at times be held responsible for the things we did *not* do. Or to use the jargon of ethicists: We are always responsible for our commissions, but occasionally we are also responsible for our omissions. Other terms frequently used to label such morally reprehensible omissions are “failure” or “neglect.”

The teacher who fails to help a struggling pupil incurs a moral risk. The mechanic who neglects to tighten the bolts incurs a moral risk. And likewise, the nurse practitioner who fails to properly sterilize the injection site, or the respiratory technologist who neglects to inform the patient of common side-effects. What sets all these examples apart from other cases of passivity is that the protagonists did not do what they were morally obligated to do. A respiratory therapist is not obligated to disclose every possible side-effect; but she is obligated to inform the patient about serious or common side-effects. A nurse practitioner is not morally obligated to verify that the manufacturer properly sterilizes each needle prior to packaging; but he is obligated to properly sterilize the injection site itself.

We can conclude, then, that every single time health care professionals decide to do something (i.e., commit an act), they are morally responsible for that decision and its consequences. But such moral responsibility does not always arise if a care giver decides *not* to act but remains passive instead. Such an omission is ethically blameworthy only if the care giver was morally obligated to act but failed to do so.

How does this translate to the domain of life-sustaining medical interventions? Any time pulmonologist Dr. P. hooks up a patient to a ventilator, she thereby incurs moral responsibility for that decision and its consequences. It does not matter whether the ventilation is a standard, even routine intervention that every pulmonologist would have initiated under the circumstances. If, for example, the patient is severely harmed as a result of the ventilation, Dr. P. should feel bad about that outcome. If, for example, it becomes clear that the patient had refused the ventilation but Dr. P. had forced the treatment onto the patient, she could be liable to charges of battery. However, the situation is more complex if Dr. P decides *not* to ventilate the patient. Now the question arises whether Dr. P. was morally obligated to initiate the ventilation.

### The two necessary conditions for medical treatment

Two necessary conditions must be met before a health care professional is morally permitted to provide a treatment. Firstly, the treatment must be medically indicated. That is, the provider must conclude that given this patient’s diagnosis and prognosis, treatment X has a reasonable chance of benefitting the patient and is unlikely to cause disproportionate harm.

Secondly, the patient (or the patient’s proxy decision maker in case the patient herself is incompetent) must be informed about her diagnosis, prognosis, and the nature of treatment X, and must then consent to it. In rare circumstances, such as when an incompetent patient with a life-threatening condition is brought to the Emergency Room, the patient’s consent may be presumed. But even then it is this “presumed consent” that fulfills the second necessary condition for initiating a medical intervention.

If either of these two necessary conditions is not met, a health care provider may not provide treatment. Thus, if Dr. P. decides that ventilation of patient A will not likely relieve the patient’s symptoms or surely cause more harm than good, forgoing ventilation is not a form of neglect. Indeed, knowingly providing a treatment that is likely to be futile violates the bioethical principle of non-maleficence and may legally constitute battery if the foreseen harm actually occurs.

Suppose Dr. P. concludes instead that ventilation *is* medically indicated for patient A. She next informs her competent patient about his condition and his options, but the patient refuses the option of ventilation. Once again, a necessary condition for treatment has not been fulfilled. So the physician ethically may forgo the ventilation. Moreover, if she forces the ventilation onto the patient anyway, she may be liable to legal charges of battery.

### Passive euthanasia

As straightforward as the foregoing analysis may seem, in real life it turns out to be very difficult to withhold or withdraw a life-sustaining treatment. Many health care providers believe that *any* omission of a life-sustaining treatment is tantamount to euthanasia or at least assistance in the patient’s suicide. And health professionals are not the only ones to reason in this fashion. In one of the trials involving the American physician Jack Kevorkian (who assisted many patients in their suicides), the judge contended that “The distinction between assisted suicide and the withdrawal of life-support is a distinction without merit.” [10].

The late American bioethicist James Rachels likewise tried to defend the morality of euthanasia by equating it to the withdrawal of life-sustaining treatment. To do so, he used a thought experiment about an uncle who is supposed to keep an eye on his little nephew while the latter is taking a bath. If the uncle pushes the kid under water and drowns him, surely he is guilty of murder, Rachels contends. Now suppose the kid falls while in the tub, hits his head, and unconsciously slips under water. If the uncle passively stands by without grabbing the kid, letting him drown instead, the uncle is equally responsible for the child’s death even though he did not actually do anything. Apparently, so Rachels concludes, the difference between being active and remaining passive has no ethical significance [11].

But the US Supreme rejected that conclusion, insisting instead that “the distinction between assisting suicide and withdrawing life-sustaining treatment.... is both important and logical; it is certainly rational” [12]. To understand why the distinction is valid indeed, we need to remember our conclusion reached earlier: Not all instances of a physician’s remaining passive amount to neglect. The physician must have failed to do what he was morally obligated to do. So was he morally obligated to provide the life-sustaining treatment?

If the health care team after a careful assessment of the patient’s condition and the patient’s own goals and interests is convinced that a particular life-sustaining treatment will do more harm than good, it should not attempt such treatment, even if the patient will surely die without. The team, then, is *not* like the second uncle in Rachels’s thought experiment who stood by idly when he could have simply grabbed the kid out of the water. This health care team cannot grab the patient out of death’s clutches. It is more akin to the sea captain who sees one of his sailors being swept overboard by a raging storm; jumping after him will be pointless.

Similarly, if the patient refusing the life-sustaining treatment is competent, one of the two necessary conditions for treatment discussed above is not fulfilled and hence the patient’s health care providers are not ethically permitted to start the treatment. It may be the case that the patient is refusing the treatment in an attempt to end his life. But even if the refusal is suicidal, that does not mean the health care team is assisting the patient in his suicide. The team simply has no ethical mandate to start the life-sustaining treatment when a competent patient refuses the treatment. For sure, the team members should inform the patient, counsel him, negotiate, and use any other respectful means to get the patient to at least try a life-sustaining treatment that is likely to be effective and unlikely to cause severe side-effects. But if a competent patient persists in his refusal, the health care team has no longer a choice in the matter, must abstain from the refused treatment, and hence cannot be responsible either for the patient’s subsequent death.

So we can conclude that in order for health care providers to be liable to charges of passive euthanasia, they must be neglectful; they must have failed to provide a treatment that is both medically indicated and consented to by the patient.

But there is more. For the team’s abstention of treatment to qualify as passive euthanasia, it must first qualify as “euthanasia.” That is to say, the decision to abstain from further treatment must be aimed at securing the patient’s death. Consider the case of Karen Quinlan again. After she had been in a PVS for more than a year and after much legal wrestling, it was finally decreed that the ventilator should be removed. But upon weaning Ms. Quinlan from the ventilator, she continued to breathe on her own (and actually lived for nine more years until dying from a pneumonia). Now if the health care providers, surprised by her survival, had exclaimed “That was not supposed to happen! We had planned for her to die,” this might have cast doubt on their intentions. But in the absence of evidence to the contrary, it is much more reasonable to assume that health care professionals who conclude that a treatment is doing more harm than good and hence needs to be forgone, do so out of the humble acknowledgment of and acquiescence in their own limits and in the limits of modern medicine more in general.

### The difference between withholding and withdrawing

So far, we have not distinguished between withholding and withdrawing a treatment. But as a matter of fact, it appears to be much more difficult for health professionals and family members to agree to the withdrawal of a life-sustaining intervention. Once a patient has been hooked up to life-sustaining technologies, those tend to acquire the status of a patient’s own organs which it would be wrong to cut out. Consider artificial nutrition and hydration. We seem to forget that it was us who surgically made an artificial opening in the patient’s abdominal wall and inserted a plastic tube through which factory-produced nutrients are being pushed by a man-made machine running on electricity supplied by the nearby power plant. There is nothing natural about this manner of consuming a meal. And yet the artificial feeding tube has somehow acquired the status of an umbilical cord that may not be cut.

From an ethical perspective the same two necessary conditions outlined earlier for medical treatments apply equally to the initiation of treatments and the continuation thereof. A health care provider needs to have both a medical indication and a consent to start treatment; and she likewise can only continue the provision of treatment when and as long as that treatment is still medically indicated and the patient is continuing to consent to its provision.

If, for example, the expected benefits of a treatment do not materialize and the harmful side-effects are more serious than expected, that treatment must be discontinued. It would be immoral to tell the patient “we are sorry that the treatment turned out to be harmful, but since we started it, it now must be continued.” Likewise, if a competent patient withdraws his earlier consent for treatment, it would be immoral to tell the patient: “Sorry, but you should not have consented to us starting the treatment; your earlier consent now allows us to continue forcing it upon you.”

In fact, the withholding of treatment is morally more risky that the withdrawing thereof. At least if a treatment was tried for a while and then shown not to benefit the patient, there is clear evidence that it is not medically indicated anymore. But decisions to withhold treatments prior to a trial period are always based on predictions only.

Finally, if a treatment of uncertain benefit is tried for a while, but the hoped-for benefits don’t materialize, its withdrawal is not a new decision that must be ethically justified. Rather, it is the termination of a clinical experiment that failed.

## Clinical decision making - Part II

### The case of Mr. French

Mr. Peter French is a 62 year old man who had cardiac surgery two weeks before but is still in the Intensive Care Unit because the medical team has been unable to wean him off mechanical ventilation. The patient has a past history of both fibrotic and obstructive lung disease related to working in the stone cutting industry, as well as a 45 year heavy smoking history. Prior to surgery, the patient had significant dyspnea with exertion and was unable to walk up more than one flight of stairs without having to stop and rest. His exercise was also limited by chest pain, secondary to coronary artery disease. The preoperative medications included both inhaled bronchodilators and cardiac vasodilators. But preoperative pulmonary function studies showed a severe combined restricted and obstructive pulmonary dysfunction with minimal improvement after bronchodilators.

Extubation has been attempted twice with rapid deterioration in arterial blood gases, necessitating reintubation of the patient within six hours. The medical team is not in agreement on how to proceed. On the one hand, the team has not been able so far to identify any potentially reversible causes of the patient’s ventilator dependency. On the other hand, the patient appears stable as long as he is ventilated. To add to the complexity, Mr. French himself, though unable to speak, has made it known that he wants the ventilator removed. His daughter, in contrast, wants everything possible done and argues that her father is depressed and suicidal, and the removal of the ventilator would be tantamount to assisting in that suicide. His son, finally, maintains that a willingness to remove the ventilator should imply willingness on the part of the health care team to end the patient’s life rather than await a slow death from hypoxia.

As we pointed out earlier, in everyday life people are more likely to be held responsible for what they *did*, than for what they did *not* do. If what they did was good, we praise them; if it was bad, we blame them. But we don’t typically praise them for not doing bad things, nor blame them for the many good things they could have done but did not. After all, the number of good things that a person can do in theory, far exceeds the average person’s capacity for doing good. So it is only in specific situations when a person has an established moral obligation to do some good thing that we will blame him for his omission to act accordingly. Hence, in everyday life doing something is morally more risky than remaining passive.

In contrast, in the world of health care, it is the forgoing of treatment that tends to generate more moral trepidation on the part of the care givers, desperation for the patient, dissension among family members, and severe anxiety for the institution’s risk manager. There seems to be moral safety in at least doing something. And at times, even legislatures and courts support this type of medical activism.

But from a strictly ethical perspective it is the (continued) *provision* of a treatment that must be justified, not withholding or withdrawing a medical intervention. For example, only if a respiratory therapist wishes to initiate an intervention should he be ready to justify that action, proving that the intervention is medically indicated and that consent for the treatment has been obtained from the patient. A therapist does not need to justify why he is *not* offering to a patient a treatment option that is deemed futile (though he may have to explain to the patient what renders this particular intervention futile). From an ethical perspective, the default position is “do *not* treat.”

As straightforward as these theoretical considerations are, they do not necessarily relieve the angst at the bedside. The clinical decision making process is often rendered more complicated still when and because it is not clear which medical treatments can benefit the patient and which have become futile. To make matters worse, in many such instances the patient is no longer competent to make decisions, has not left a clear advance directive, and family members disagree about which treatments to consent to and which to refuse on behalf of the patient. These different factors then become all mixed-up, yielding an emotionally volatile situation that defies a calm and mutually agreeable resolution.

The scenario sketched above exemplifies such a volatile situation. Although the case description is too sparse to enable a comprehensive ethical analysis, it does lend itself to illustrate how a systematic application of ethical considerations can promote a morally sound resolution.

### Clarifying the intent

The case of Mr. French is particularly charged because of the references made (by the daughter) to physician assistance in suicide (PAS) and even (by the son) to euthanasia. These two practices differ in the degree to which the patient himself or the health care provider is responsible for the final outcome, that is, the patient’s death [13]. But they are similar in that both practices are aimed at that outcome. The patient’s death is not simply an accident, nor even an undesired but tolerated side-effect. Both in PAS and euthanasia, the patient’s death is intended and the interventions are specifically chosen to bring about that outcome.

However, Mr. French’s son is mistaken in his contention that every decision to withhold a life-sustaining treatment equates or should equate willingness to end the patient’s life. As explained in the first part of this article, a willingness on the part of care givers to let go does not logically equate a willingness to bring about the patient’s death. The former reflects an acceptance of one’s limited powers as a healer in the face of human frailty and mortality; the latter reflects a desire to exert power over the situation and a belief that an enacted death is morally preferable to a natural death.

There certainly are instances in which withholding or withdrawing a life-sustaining treatment constitutes neglect and may be tantamount to assisting in suicide or even euthanasia. But the daughter is mistaken in concluding that withdrawal of the ventilator of Mr. French necessarily qualifies as such. Or at least the facts as presented do not allow for such a conclusion. The more likely interpretation is that a third and final attempt at withdrawing the ventilator would be motivated by the health care team’s respect of the patient’s autonomous decision to forgo further ventilation, and not be aimed at bringing about the patient’s death.

In order to increase the odds of achieving consensus among all involved about the overall goals of treatment and care, it would be prudent for the medical team to meet with the patient and his children to explicitly discuss these goals. Except in those few countries where euthanasia is legal, it should be easy to reach consensus that the principal goal of the care is not to end the patient’s life but shall only be to make the patient’s remaining time of life the best possible time of life. Exactly how much time of life can be gained through medical interventions and at what price has yet to be ascertained. But the patient’s death shall not be anybody’s goal.

### Determining futility

Having now agreed on the broad goal of the care to be rendered, the medical team can begin to determine which medical interventions are indicated for Mr. French, and which would be futile. Any treatment that is indicated should be presented to Mr. French for his consent. But any intervention that is deemed futile should be withheld or withdrawn.

We stipulated earlier that a treatment is medically indicated, as opposed to futile, if it is likely to benefit the patient. This definition contains two terms that health care professionals use routinely but that are actually very difficult to define and ascertain. “Likelihood” may seem a simple statistical concept, representing the odds that a particular outcome will come about. But what is the ethical relevance of such odds? Should a physician abstain from any treatment that has only a 49% chance of benefitting the patient because the treatment is more likely to cause harm than good? Would a patient be a poor steward of his God given life if he refuses a treatment that has a 10% chance of extending his life? What if the odds are 25%? Or 5%?.

Equally difficult is it to define the concept of “benefit.” To benefit a patient literally means to do good for the patient. But this presumes that we know what is good for the patient. Clearly, an assessment of what is for the good of the patient is not a scientific, value neutral judgment. But neither are derivative assessments, such as what is normal or abnormal, what is healthy or unhealthy, physiological or pathological. None of these terms is value neutral.

In most clinical scenarios, we can use these terms without actually defining them because the goals of treatment are clear and mutually agreeable to both the patients and their caregivers. But when health can no longer be achieved, when life’s end is nearing, when suffering is severe and the means to relieve it have themselves nasty side-effects, it is suddenly no longer self-evident what is in the patient’s best interests, neither to the health care professionals, nor to the patients’ family members or even the patients.

Thus, it would behoove the team to very carefully assess the specific goals of continued treatment of Mr. French. Is the patient tired of living with his disabilities and no longer able to muster the mental energy to adjust his life yet again to still more restrictions on his physical functioning? Does he dread being held hostage for the remainder of his life to a breathing machine? Is he angry and upset about the disappointing outcome of the surgery but deep down longing for a few more years on this earth?

Upon achieving more insight into the specific goals, the medical team can determine the available means to achieve those goals, how likely the goals can be achieved with those means, and what are the unintended but likely and harmful side-effects of the various treatment options. Armed with a carefully considered set of treatment options, the team might be able to provide Mr. French with a third alternative between more of the same, or death. And if nothing else, an honest and humble admission of the team’s own moral discomfort with either of the two aforementioned options may lead to some sort of negotiated deal between patient and team that buys the team more time to develop alternative options, and assures the patient that the medical team is ready to let him go when the extension of the treatment trial has run its course without yielding the improvement that everybody had been hoping for.

### Competence and consent

Almost as difficult, or at least as contentious, as defining and determining futility is the definition and determination of patient decision making incompetence. As long as patients agree to proposed treatments, medical providers tend to assume that patients are competent – as if the mere fact that a patient agrees with his physician proves that the patient is competent. Conversely, if the patient does not consent or withdraws an earlier consent, almost immediately questions will be raised about his competence. This equation of a patient refusal with patient decision making incompetence makes a mockery of patient autonomy and signals a return to old-fashioned paternalism.

But even if we grant that this is not how patient decision making incompetence should be ascertained, that does not tell us how it *should* be done. As recent research has made clear, there is no consensus among either ethical, legal, or psychological experts about the definition and determination of this key concept in medical ethics [14]. There is even less agreement about the determination of reliable alternate sources of consent for treatment after patients have been determined incompetent.

In the case of Mr. French, it is possible that the unexpected and very disappointing outcome of the cardiac surgery has caused a depression. It would be important for the health care team to assess whether such a depressive mood is clouding the patient’s free decision making abilities to such an extent that he is incompetent to provide consent. But unless and until Mr. French has been proven incompetent, he should be considered competent.

Also, if Mr. French were to be found incompetent, this does not suddenly justify continuing the ventilation. The team would still need to get the consent for continued treatment from somewhere. It is not at all clear how or from whom the consent for continued ventilation can be obtained in this case. And it is certainly possible that a surrogate decision maker would likewise refuse continued ventilation, arguing that Mr. French, had he been competent, would himself have refused.

For a consent to treatment to be valid, it must be issued by a competent patient or surrogate, and it must be an informed consent. Although it is not the case that a patient’s refusal of treatment is valid only if it is an informed refusal, the care team should always strive to properly inform the patient about his condition, prognosis, and options. The facts of the case scenario do not tell us whether Mr. French, while competent, has been properly informed and carefully considered the information provided. For example, does he know what options are available to him besides a life in the ICU? Has he met other patients who have learned to live with similar disabilities? Also, is he being protected against too much information, specifically distracting and confounding information provided by his two quarreling children?

## Conclusions

When death comes knocking on the door, patients, family members, and health care providers alike shift into rescue mode [9]. We want to do something, try another diagnostic tool, attempt another medication, enroll in a clinical trial, anything. Add to this drive to act the widespread belief that medicine can literally achieve miracles, and it is easy to understand why it is so difficult for all involved to withhold a life-sustaining treatment and even more difficult to withdraw one that has already been started.

In these situations it behooves care givers to remember that, ethically, the default position is *not* to treat. It is the initiation or continuation of medical interventions that must be ethically justified. Such interventions, including life-sustaining medical treatments, are ethically justified *only* if *both* of the following necessary conditions have been met: (1) the treatment must be medically indicated; and (2) there must be a consent for the indicated treatment.

As in the case of Mr. French, it is often difficult to ascertain which medical interventions are indicated and which have become futile. Moreover, a risky intervention with a very small chance of success may be beneficial to one patient but futile for another. But once it has been determined that a particular intervention is not indicated, it should not even be offered to the patient (though it may necessary to explain to the patient why the intervention is futile). And if an intervention was initiated in the belief that it was going to benefit the patient, but has since been found to do more harm than good, it should be withdrawn. Such forgoing of a futile medical intervention is not tantamount to passive euthanasia but acquiescence in the mortality of human beings and the limits of medical power.

Because the patient’s good is not a value-neutral, statistical concept, care givers have to involve their patients in order to determine what treatments will benefit them and what treatments, hence, are indicated. But since the patient is the one undergoing the medical intervention, the patient always gets the final say. Treatments, even those that are clearly indicated, cannot be started or continued unless and only as long as the patient consents.

Mr. French’s refusal of continued ventilation does not prove his incompetence. Unless Mr. French is diagnosed as suffering from a clinical depression that clouds his decision making capacity, we have to assume he is competent. Hence, the members of the medical team are ethically required to abide by his refusal of further ventilation. That does not bar the team members from expressing their own moral discomfort or recommending a longer trial period, as long as such “pressure” is exerted in a manner that fully respects the patient’s autonomy. But if Mr. French persists in his refusal, the care team has no choice but to discontinue the ventilation, and then seek to provide the best palliative support possible for Mr. French.

Finally: even if Mr. French were to reveal that by refusing continued ventilation, he is actually seeking to end his own life, this does not mean that the physicians likewise seek to bring about the patient’s death if they remove the ventilator. They need the patient’s consent to treat or continue treatment, and they no longer have it. Stepping back, then, does not amount to assisting in the patient’s suicide, but is a token of respect for the patient’s autonomy.

## Competing interests

The authors declare that they have no competing interests.
